# Dead or Alive; or Does It Really Matter? Level of Congruency Between Trophic Modes in Total and Active Fungal Communities in High Arctic Soil

**DOI:** 10.3389/fmicb.2018.03243

**Published:** 2019-01-08

**Authors:** Magdalena Wutkowska, Anna Vader, Sunil Mundra, Elisabeth J. Cooper, Pernille B. Eidesen

**Affiliations:** ^1^Department of Arctic Biology, The University Centre in Svalbard (UNIS), Longyearbyen, Norway; ^2^Department of Arctic and Marine Biology, Faculty of Biosciences, Fisheries and Economics, UiT - The Arctic University of Norway, Tromsø, Norway; ^3^Section for Genetics and Evolutionary Biology (EVOGENE), Department of Biosciences, University of Oslo, Oslo, Norway

**Keywords:** below-ground processes, fungal trophic mode, fungal functional group, snow regime, arctic vegetation, snow fences

## Abstract

Describing dynamics of belowground organisms, such as fungi, can be challenging. Results of studies based on environmental DNA (eDNA) may be biased as the template does not discriminate between metabolically active cells and dead biomass. We analyzed ribosomal DNA (rDNA) and ribosomal RNA (rRNA) coextracted from 48 soil samples collected from a manipulated snow depth experiment in two distinct vegetation types in Svalbard, in the High Arctic. Our main goal was to compare if the rDNA and rRNA metabarcoding templates produced congruent results that would lead to consistent ecological interpretation. Data derived from both rDNA and rRNA clustered according to vegetation types. Different sets of environmental variables explained the community composition based on the metabarcoding template. rDNA and rRNA-derived community composition of symbiotrophs and saprotrophs, unlike pathotrophs, clustered together in a similar way as when the community composition was analyzed using all OTUs in the study. Mean OTU richness was higher for rRNA, especially in symbiotrophs. The metabarcoding template was more important than vegetation type in explaining differences in richness. The proportion of symbiotrophic, saprotrophic and functionally unassigned reads differed between rDNA and rRNA, but showed similar trends. There was no evidence for increased snow depth influence on fungal community composition or richness. Our findings suggest that template choice may be especially important for estimating biodiversity, such as richness and relative abundances, especially in Helotiales and Agaricales, but not for inferring community composition. Differences in study results originating from rDNA or rRNA may directly impact the ecological conclusions of one’s study, which could potentially lead to false conclusions on the dynamics of microbial communities in a rapidly changing Arctic.

## Introduction

Species loss is a major concern in ecosystem functioning (Cardinale et al., 2012). Amplicon sequencing of DNA extracted from environmental samples has become a common tool for species detection (Bohmann et al., 2014; Bass et al., 2015; Barnes and Turner, 2016). Since only a small fraction of microbes, including fungi, can be cultured in the laboratory, monitoring of these species relies heavily on analysis environmental ribosomal DNA (rDNA) (Creer et al., 2016). Microbes are embedded in multi-species assemblages that closely interact with each other on small spatial scales; genomic methods based on rDNA used to describe their characteristics, such as taxonomic diversity (Konopka, 2009). Despite tremendous advancements in molecular methods, estimating biodiversity and community composition in many groups of organisms, such as fungi, remains challenging (Costello, 2015; Hawksworth and Lücking, 2017).

Critical assessment of results, recommendations and best practices for rDNA metabarcoding is still debated (Goldberg et al., 2016; Shelton et al., 2016). Methodological biases may heavily influence fungal study outcomes; this includes bypassing detection of certain taxonomic groups by choosing particular marker genes (Schoch et al., 2012) or even marker gene regions (Blaalid et al., 2013). In spite of these methodological limitations, a growing body of evidence suggests that the choice between nucleic acid template, namely rDNA, and its transcribed product rRNA, may provide inconsistencies. This is due to the fact that rDNA does not have to correspond with the presence of living cells in the environment (Anderson and Parkin, 2007; Pedersen et al., 2015; Carini et al., 2016). Physicochemical properties of the environment, such as cold temperatures or soil particle adsorption properties, can enhance preservation of DNA from dead organisms (Ogram et al., 1988; Saeki and Kunito, 2010; Saeki et al., 2011). It was recently shown that using rRNA as sequencing template was superior to rDNA in detecting live bacterial cells in water (Li et al., 2017). The turnover rate of DNA is expected to be much slower in soil than in water (Thomsen and Willerslev, 2015). Thus, rDNA metabarcoding of soil samples has a high risk of being biased by dead material.

Risk of bias in biodiversity assessment from dead material is particularly high in samples of soil dwelling organisms from cold climate regions, In the Arctic, lower temperatures slow down the rate of microbial decomposition and cells or extracellular DNA may freeze within permafrost (Gilichinsky et al., 1995; Soina et al., 1995). Old organic material can later intermix through physical processes in the soil, such as cryoturbation, which enables soil from deeper depths to be raised to the top exposing biological material frozen many years ago (Kaiser et al., 2007). To circumvent these problems, an alternative is to use rRNA as a metabarcoding template. RNA degrades rapidly when it is no longer needed in the cell, and therefore gives information about the metabolically active cells that contribute to microbial processes (Blazewicz et al., 2013).

Species can play redundant roles in an ecosystem, therefore recent ecological studies stress targeting functional diversity in ecosystems, as opposed to biodiversity only (Louca et al., 2016; Cernansky, 2017). Many fungal species play redundant roles in ecosystem functioning by exploiting or altering the distribution of the same resources (Moore et al., 2011). In recent years some fungal studies focused on parsing operational taxonomic units (OTUs) into ecologically meaningful groups that play the same function in the ecosystem, such as trophic modes, represented by symbiotrophs, saprotrophs and pathotrophs (Nguyen et al., 2016). All of these trophic modes are important in arctic ecosystems. Saprotrophic fungi acquire their organic carbon through decomposition of dead biomass, and are important for carbon- and nutrient cycling in arctic soils (Buckeridge and Grogan, 2008; Kohler et al., 2015). Symbiotrophic fungi acquire their organic carbon through mutualistic partnerships, especially with plants. This group includes mycorrhizal fungi that play an important ecological role by supporting plant uptake of nutrients and water, notably important in arctic tundra where especially nitrogen may be heavily depleted (Gardes and Dahlberg, 1996; Timling and Taylor, 2012). Pathotrophic fungi that obtain organic carbon by harming host cells play a role in controlling other trophic levels in the ecosystem (Fodor, 2011). Previous studies have suggested that altered climate can change soil carbon balance, affecting vegetation composition through the influence of pathotrophic fungi (Olofsson et al., 2011).

Fungi play important ecological roles in Arctic terrestrial ecosystems and current knowledge on how Arctic fungal biodiversity is shaped by climate changes remains scattered (Timling and Taylor, 2012). Investigating these fungal responses is clearly at risk of being affected by both methodological bias and bias induced by extracellular rDNA, which was estimated to contribute up to 40% of all sequences in soil samples, thus escalating observed richness and misleading conclusions about prokaryotic and fungal communities (Carini et al., 2016). Response of fungal communities to some manifestations of these changes in the Arctic, such as increased winter precipitation, were studied using only rDNA (Morgado et al., 2016; Mundra et al., 2016b; Semenova et al., 2016). Thus, none of these studies discriminated between metabolically active cells, dead matter, spores or relict rDNA.

In this case study we assess how the choice of metabarcoding template (rDNA vs. rRNA) influences the fungal soil community retrieved from soil samples under different environmental conditions. We sampled soil in an experimental setting of snow fences mimicking increased winter precipitation in two distinct vegetation types: heath and meadow (Morgner et al., 2010). Then we sequenced ITS2, analyzing rDNA and rRNA-based metabarcoding data separately. Our main aim was to determine whether results and ecological conclusions based on rDNA and rRNA metabarcoding templates were congruent. We analyzed the data in relation to fungal trophic modes, here defined as symbiotrophs, saprotrophs and pathotrophs (Nguyen et al., 2016). We also compared rDNA and rRNA results in relation to community composition (1) and OTU richness (2). Finally, we looked into how various edaphic variables influenced community composition as well as OTU richness for different fungal trophic modes. Incongruent results between the two metabarcoding templates at any of these levels may potentially point toward types of analyses that can create a misleading picture of the ecosystem.

## Materials and Methods

### Sampling Site, Experimental Setup, and Sample Collection

Snow fences established in Adventdalen, Svalbard (78°10 N, 16°02-16°05 E), altered snow regime since winter 2006/2007, creating approximately 1 m deeper snow in treatment plots compared to controls (Morgner et al., 2010; Supplements 1 and 1a). Deep snow regime altered annual patterns of two important physical variables for soil dwelling microorganisms: soil moisture content and temperature (Cooper et al., 2011). Fences were established in blocks of 3 fences with 2 blocks per vegetation type: heath and meadow. Deep snow regime generally had higher soil nutrients (NO_3_^-^N, NH_4_^+^N, and K) than ambient (Semenchuk et al., 2015; Mundra et al., 2016b).

Sampling took place on 28 and 30 of August 2012, simultaneously with a study focusing on Bistorta vivipara root associated communities from the same sites (Eidesen, unpublished data). After an individual B. vivipara plant with its whole root system had been excavated using a small shovel, two soil samples were collected, filling 2 ml cryo-tubes, from opposite sides of the resulting hole. The soil samples, procured from 5 to 10 cm depth with a sterile spatula, were immediately frozen in liquid nitrogen. In total 96 samples were collected; 2 holes × 2 soil samples × 2 snow regimes × 3 fences × 2 blocks × 2 vegetation types. Edaphic parameters were measured according to protocols described in Mundra et al. (2015b). To minimize the effect of small-scale spatial variability the two primary samples from the same hole were combined prior to analyses, resulting in 48 true samples (referred to in the remaining text).

### Obtaining rDNA and rRNA Sequences

rRNA and rDNA was co-extracted from 1 to 2 g of frozen soil using the PowerSoil Total RNA Isolation Kit (MO BIO Laboratories, United States) and PowerSoil DNA Elution Accessory Kit (MO BIO Laboratories, United States), both according to manufacturer’s instructions. Complementary DNA (cDNA) was synthesized using the Maxima First Strand cDNA Synthesis Kit for RT-qPCR, with dsDNase (Thermo Fisher Scientific, United States) following the manufacturers’ instructions, except that a 5 min incubation step was used for DNase treatment. After DNase treatment, a 1 μl subsample was used as a no-RT control during subsequent PCR amplification. All no-RT controls were negative, showing that DNase treatment had been successful and that cDNA amplification during RT-PCR was due to rRNA template.

PCRs and library preparations was carried out for rDNA and cDNA as described in Mundra et al. (2016b), using the primers fITS7a (Ihrmark et al., 2012) and ITS4 (White et al., 1990) to amplify the internal transcribed spacer 2 (ITS2) region of the nuclear ribosomal DNA, using 1 μl of DNA/cDNA as templates. The protocol for library preparation is described in Mundra et al. (2016b). The multiplexed samples were paired-end sequenced (2 × 300 bp) on an Illumina MiSeq sequencer at ACGT Inc, Wheeling, United States.

### Bioinformatic Analysis of Sequencing Data

The bioinformatic analysis of Illumina sequences followed the pipeline described by Bálint et al. (2014) with minor modifications. A total of 8,413,098 paired-end sequenced reads were filtered using a perl script (supplemented in Bálint et al., 2014). The remaining 7,779,879 high quality paired-end sequenced reads (high quality score > 26) were assembled in PANDAseq 2.6 (Masella et al., 2012). After quality filtering and assembly, 23 rDNA and 19 rRNA samples were retained in the analyses. Sequences with primer artifacts were removed with a python script (supplemented in Bálint et al., 2014), prior to reorientation using fqgrep 0.4.4^1^ and the fastx_reverse_compliment function from Fastx Toolkit 0.0.14^2^ to reverse sequences identified as oriented in the 3′–5′ direction containing 7,028,992 reads. To demultiplex sequences with variable length barcodes we used the split_library.py script in Qiime 1.9.1 (Caporaso et al., 2010), retaining sequences of 200–500 bp, allowing for 1bp primer mismatch, and maximum length of homopolymer run equal to 8.

5,184,214 demultiplexed sequences were then sorted by length in a range and dereplicated, before sorting groups by size, excluding those containing less than five sequences (Nguyen et al., 2015) in Vsearch 2.7.1 (Rognes et al., 2016). Using 0.97 sequence similarity threshold, 2185 operational taxonomic units (OTUs) were picked by the cluster_otus function (usearch 8.1.1861; Edgar, 2010) and then 232 putative chimera sequences were removed in reference based chimera check with uchime2 (Edgar, 2016) against fungal database UNITE+INSD (Kõljalg et al., 2013; version: UNITE_public_01.12.2017). To retain only ITS2 fragments of fungal origin, sequences were filtered through ITSx v. 1.1b (Bengtsson-Palme et al., 2013), leaving 1473 representative sequences. To further exclude non-fungal sequences we used local blast search (blast+ 2.6.0) against the nucleotide NCBI database (updated 13.12.2017) and parsed these results in MEGAN Community Edition 6.5.10 (Huson et al., 2016) as described in Bálint et al. (2014). Unclustered sequences were mapped against representative sequences identified as fungal in MEGAN to obtain an OTU abundance table, which then was rarefied to 42,488 reads per sample with single_rarefaction.py in Qiime 1.9.1 (Caporaso et al., 2010). The level of rarefaction was set based on output from the demultiplexing step. Several samples with very low read numbers (0–2870 reads), were removed during this step, based on the assumption that these samples had failed during the sequencing reaction. The distribution of failed samples was random, and although leading to a lower number of total samples in the study and hence lower statistical power, should not affect the conclusions of our study.

The final OTU table with rDNA and rRNA samples contained 837 OTUs. Since correct identification of species determines more precise functional assignment, the final taxonomy was assigned by querying representative sequences against the curated fungal database UNITE. In cases where we did not get a blast hit, taxonomy was assigned using the NCBI-NT database. Eight OTUs were assigned as Rhizaria (all as unidentified class of Cercozoa). We decided to keep these due to the fact that they remained in the dataset through two steps of removing non-fungal OTUs (see above). OTUs were categorized into trophic modes: symbiotrophs, saprotrophs and pathotrophs (Supplement 1) using FUNGuild (Nguyen et al., 2016). OTUs assigned to multiple trophic modes, as well as OTUs with taxonomic assignment that precluded accurate assignment to a trophic mode, were marked as “unassigned.” The OTU table was divided into separate matrices for rDNA and rRNA, which were analyzed separately for the rest of the study.

### Statistical Analysis

Statistical analysis was performed in R v3.4.4 (R Core Team, 2018). All described statistical analyses were performed in parallel for both rDNA and rRNA.

### Community Composition

Global non-metric multidimensional scaling (GNMDS; Kruskal, 1964) was used to analyze dissimilarity matrixes within rDNA- and rRNA-based community compositions of the samples containing all OTUs, symbiotrophs, saprotrophs and pathotrophs separately, on presence-absence OTU tables using the Jaccard dissimilarity index. In ordination analyses we used presence-absence data to avoid biases associated with possible differences in RNA copy number. The ordination analyses were performed following Liu et al. (2008). Loss of data during sample preparation and data processing allowed a direct comparison of only nine extracted pairs of rDNA and rRNA samples, which was tested by Mantel’s test with 9999 replications (ade4 package 1.7-11, Chessel et al., 2004). Possible relationships among community composition, edaphic variables and experimental factors were investigated. The envfit function in vegan package (v. 2.5-2; Oksanen et al., 2018) was used for multiple regressions of edaphic variables and vegetation type. Permutational multivariate analysis of variance (PERMANOVA) implemented as adonis function in vegan package were used to assess the effect of vegetation type, snow regime, and their possible interaction. In the PERMANOVA, we accounted for spatial variability observed in earlier studies (Mundra et al., 2015a, 2016a,b) by selecting blocks of fences as a random source of variation. Strength of relationships between GNMDS axis, edaphic variables and experimental factors were assessed based on R2 coefficients of determinations and *P*-values.

### OTU Richness

OTU richness, as number of OTUs per sample, was calculated using specnumber function in vegan package. We used linear mixed effects models (lmer function in lme4 package; Bates et al., 2015) to test if there were any effects of experimental factors (nucleic acid, snow regime and vegetation type) on richness of all fungi, symbiotrophs and saprotrophs. Random effects reflected the experimental design where blocks of fences and fences are nested in the design. *P*-values were calculated in Anova function from car package (v. 3.0-0; Fox and Weisberg, 2011). In some cases, components of random variance collapsed to 0, meaning that our data were not sufficient to support a model with this level of complexity. A linear model without fitting random factors gave the same estimations, but slightly increased the values of statistical significance of the results.

## Results

### Assigned OTUs

In our analysis we retained 42 samples (23 rDNA and 19rRNA). The rDNA and rRNA combined OTU table contained 837 OTUs which included 288 symbiotrophs, 105 saprotrophs, 34 pathotrophs, and 410 unassigned OTUs (Supplement 3). The number of OTUs assigned to each trophic mode was similar in rDNA and rRNA (Supplement 3). However, symbiotrophs, the dominant trophic mode, were relatively less represented in rRNA than rDNA reads. Both saprotrophs and unassigned reads were twice as abundant in rRNA than in rDNA.

Snow regime showed no clear influence on either community composition or richness (Table 3, Supplements 4, 5, other data not shown). The “deep snow” and “control” samples within each vegetation type were therefore pooled in the presented analyses.

### Community Composition

**FIGURE 1:**
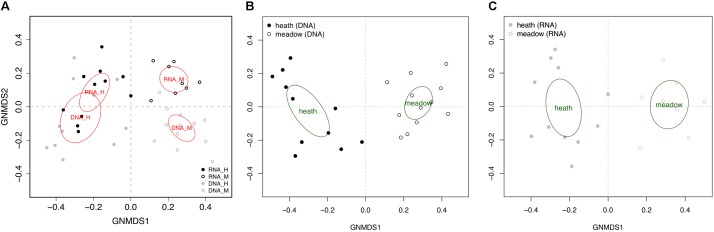
Global non-dimensional scaling of all 42 samples plotted based on presence-absence table that included 837 OTUs **(A)** and according to template (**B**, rDNA – 23 samples; **C**, rRNA – 19 samples) and vegetation type (H, heath; M, meadow).

GNMDS based on the matrix of all OTUs showed a similar overall trend of community composition for rDNA and rRNA (Figure 1). Direct comparison of rDNA and rRNA-derived dissimilarity matrices obtained from 9 co-extracted samples showed a strong correlation between the two (Mantel test observed value: 0.73, *p* < 0.001). Fungal community composition based on all OTUs was primarily divided according to vegetation types: heath and meadow, both for rDNA and rRNA (Figure 1). Separate GNMDS analyses of rDNA and rRNA for symbiotrophs and saprotrophs showed the same overall trends, with vegetation type as the main driver in shaping their community compositions (*r*2 = 0.27–0.66 with *p* = 0.004 or less).

The two vegetation types, heath and meadow, differed in edaphic parameters (Supplement 2). These edaphic variables fitted onto GNMDS (all OTUs) revealed pH as a significant explanatory variable (Table 1), but with different explanatory value depending on template (rDNA or rRNA) and trophic mode. While pH had the highest and dominant explanatory value in all analyses based on rDNA (from *r*2 = 0.77 in all OTUs and symbiotrophs; Table 1), other variables tended to explain as much variability in the rRNA dataset (especially organic matter content, as well as the connected nitrogen and carbon contents). Carbon/nitrogen ratio was an important edaphic variable for explaining rDNA-derived community composition (*r*2 = 0.27–0.39), but not at all for rRNA (*r*2 = 0.03–0.06).

**Table 1:** Edaphic variables and vegetation type as a factor fitted into global non-dimensional scaling of all 23 rDNA samples and 19 rRNA samples (plotted based on presence-absence matrixes that included all OTUs, symbiotrophs, saprotrophs, and pathotrophs).

	rDNA		rRNA r2	
		
	r2	Pr( > r)	r2	Pr( > r)
**All_OTUs**				
pH	0.77	0.001^***^	0.67	0.001^***^
Moisture	0.12	0.245	0.16	0.248
Conductivity	0.22	0.062 .	0.36	0.028^*^
Organic matter	0.12	0.256	0.69	0.001^***^
Total nitrogen	0.17	0.144	0.61	0.001^**^
Carbon	0.13	0.239	0.64	0.001^**^
Carbon/nitrogen ratio	0.39	0.011^*^	0.03	0.789
**Symbiotrophs**				
pH	0.77	0.001^***^	0.63	0.002^**^
Moisture	0.12	0.277	0.13	0.326
Conductivity	0.22	0.073 .	0.36	0.022^*^
Organic matter	0.12	0.246	0.65	0.001^***^
Total nitrogen	0.17	0.131	0.50	0.005^**^
Carbon	0.13	0.214	0.49	0.007^**^
Carbon/nitrogen ratio	0.39	0.007^**^	0.06	0.599
**Saprotrophs**				
pH	0.58	0.001^***^	0.60	0.002^**^
Moisture	0.10	0.332	0.17	0.242
Conductivity	0.10	0.340	0.11	0.356
Organic matter	0.10	0.337	0.58	0.001^***^
Total nitrogen	0.25	0.052 .	0.43	0.015^*^
Carbon	0.19	0.115	0.54	0.002^**^
Carbon/nitrogen ratio	0.27	0.048^*^	0.06	0.630
**Pathotroph**				
pH	0.14	0.224	0.03	0.807
Moisture	0.07	0.457	0.01	0.931
Conductivity	0.01	0.916	0.08	0.500
Organic matter	0.01	0.940	0.04	0.715
Total nitrogen	0.01	0.957	0.04	0.757
Carbon	0.01	0.902	0.03	0.786
Carbon/nitrogen ratio	0.03	0.735	0.01	0.918


*Signif. codes: “^∗∗∗^” 0.001 “^∗∗^” 0.01 “^∗^” 0.05 “.” 0.1 “ ” 1.*

Community composition of pathotrophs showed distinct trends in regards to clustering in GNMDS and response to edaphic variable, when rDNA and rRNA were compared, while patterns observed in symbiotrophs and saprotrophs were more similar to each other. The 95% confidence intervals on GNMDS plots showed partial (in rDNA) or total (in rRNA) overlap in meadow and heath. Furthermore, no edaphic variables could explain pathotrophic community composition (*r*2_DNA_= 0.01–0.14 and r2_RNA_= 0.01–0.08) with statistical significance (*p* > 0.224).

### OTU Richness

**FIGURE 2:**
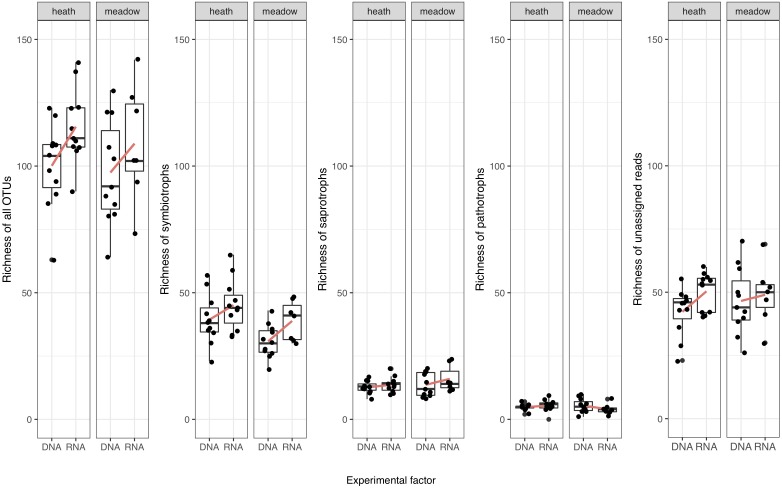
Richness of detected fungal OTUs in meadow and heath (without 2 outliers). Red lines connect mean values.

Since richness analyses are sensitive to outliers, after initial plotting of these values for all samples, we decided to remove the two highest values (one from each metabarcoding template) that were abnormally high (177 OTUs in rDNA and 159 OTUs in rRNA). Mean richness was higher in rRNA, especially in heath (Figure 2 and Table 2). The same trend was seen in symbiotrophs and unassigned reads, but neither in saprotrophs or pathotrophs (Figure 2 and Tables 2, 4).

**Table 2:** Richness of detected fungal OTUs in a snow fence experimental setup.

	n	μall ± Sd	μ_Symbio_ ± Sd	μ_sapro_ ± Sd	μ_patho_ ± Sd	μ_unassign_±Sd
DNA_H	11	100.1 ± 17.1	39.4 ± 9.8	12.6 ± 2.5	4.8 ± 1.3	42.3 ± 9.4
RNA_H	11	115.5 ± 14.6	45.1 ± 10.2	13.6 ± 3.0	5.5 ± 2.4	50.4 ± 7.5
DNA_M	12	104.1 ± 30.2	31.4 ± 6.6	15.2 ± 6.9	6.3 ± 3.8	50.3 ± 18.0
RNA_M	8	115.1 ± 27.8	41.2 ± 9.9	16.9 ± 5.5	4.3 ± 2.1	51.8 ± 13.6
DNA_M (no outliers)	11	97.5 ± 20.6	30.8 ± 6.6	13.6 ± 4.6	5.5 ± 2.7	46.5 ± 13.2
RNA_M (no outliers)	7	108.9 ± 23.1	38.6 ± 7.8	16.0 ± 5.3	4.0 ± 2.1	49.0 ± 12.0


*n, number of samples; H, heath; M, meadow; μ, mean richness for all (μ_*all*_), symbiotrophic (μ_*symbio*_), saprotrophic (μ_*sapro*_), pathotrophic (μ_*patho*_), and unnasigned OTUs (μ_*unassign*_); sd, standard deviation.*

**Table 3:** Permutational multivariate analysis of variance (PERMANOVA, adonis function in vegan package) based on rDNA and rRNA presence-absence matrixes of all, symbiotrophic, saprotrophic and pathotrophic OTUs.

	Vegetation type		Snow regime		Vegetation X Snow	
			
	*r*2	*p*	*r*2	*p*	*r*2	*p*
All OTUs rDNA	0.16	0.133	0.04	0.247	0.04	0.102
All OTUs rRNA	0.14	0.047^*^	0.06	0.011^*^	0.05	0.374
Symbiotrophs rDNA	0.15	0.505	0.04	0.617	0.04	0.391
Symbiotrophs rRNA	0.06	0.512	0.05	0.737	0.06	0.247
Saprotrophs rDNA	0.13	0.023^*^	0.06	0.024^*^	0.05	0.047^*^
Saprotrophs rRNA	0.10	0.435	0.07	0.113	0.04	0.897
Pathotrophs rDNA	0.12	0.045^*^	0.06	0.081 .	0.06	0.114
Pathotrophs rRNA	0.08	0.424	0.05	0.402	0.06	0.459


*Vegetation type and snow regime factors were first tested in forward selection before testing for interaction. Signif. codes: 0.01 “^∗^” 0.05 “.” 0.1 “ ” 1.*

**Table 4:** Results of linear mixed models explaining richness of all OTUs, symbiotrophs, saprotrophs and saprotrophs.

Response	Fixed effects					Interaction		Random effects	
			
Richness	Intercept ± 1SE	Template ± 1SE	*p*	Vegetationl ± SE	*p*	Template x vegetation type±1SE	*P*	Fence:block ± SD	Block ± SD
All OTUs	99.7 ± 5.8	16.5 ± 7.9	0.034	-2.5 ± 8.2	0.591	-4.7 ± 12.0	0.729	13.94 ± 3.7	0
Symbiotrophs	39.0 ± 3.1	6.2 ± 3.7	0.031	-8.2 ± 4.3	0.200	1.8 ± 5.7	0.775	0	5.2 ± 2.3
Saprotrophs (Im)	12.3 ± 1.0	1.0 ± 1.6	0.548	1.0 ± 1.6	0.548	1.4 ± 2.5	0.588		
Pathotrophs (Im)	4.8 ± 0.7	0.6 ± 0.9	0.503	0.6 ± 0.9	0.503	-2.1 ± 1.4	0.015		
Unassigned	41.8 ± 3.4	9.2 ± 4.4	0.080	4.4 ± 4.8	0.916	-6.2 ± 6.7	0.410	11.36 ± 3.4	0


*In cases with all OTUs, saprotrophs and functionally unassigned reads variance of random effects, both nested (fence:block) and main (block) could not be estimated (values = 0), so in these cases we used linear model (lm) instead. Table includes all the data, excluding 2 outlier samples.*

Taking into consideration experimental (metabarcoding template choice and vegetation type) and random factors, the differences in overall OTU richness were driven by the choice of metabarcoding template, rather than by vegetation type (Table 4; rDNA < rRNA, model estimation = 16.5, *SE* = 7.9, *p* = 0.034 for the template vs. model estimation for vegetation type -2.5, *SE* = 8.2, *p* = 0.591). However, based on OTU richness for 9 pairs of co-extracted samples, we saw that the effect of metabarcoding template is important, but not statistically significant (rDNA < rRNA, model estimation = 12.3, *SE* = 8.1, *p* = 0.149 for the template).

Overall, out of 827 OTUs, there were 199 OTUs present only in rDNA- and 188 only in rRNA-derived samples. In a subset of 9 co-extracted samples 528 OTUs were detected, from which 135 OTUs were only present in rDNA- and 81 OTUs only in rRNA-based results.

### Relative Abundance of Reads

**FIGURE 3:**
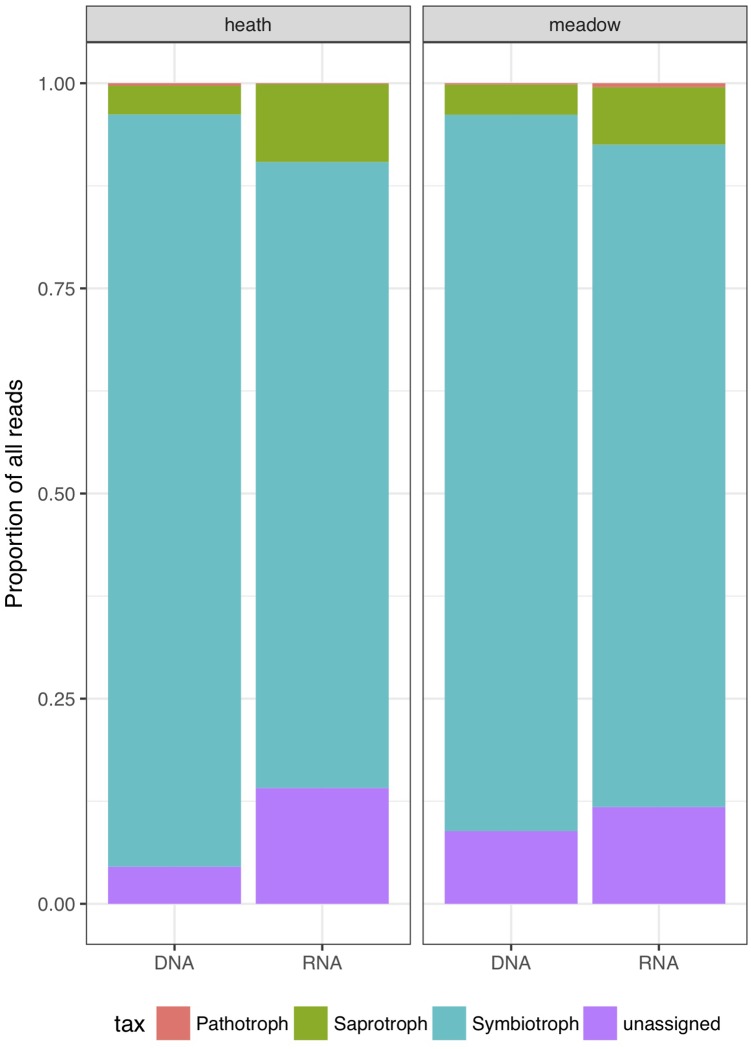
Relative abundances of all reads divided into trophic modes (saprotrophs, symbiotrophs, pathotrophs and functionally unassigned OTUs).

**FIGURE 4:**
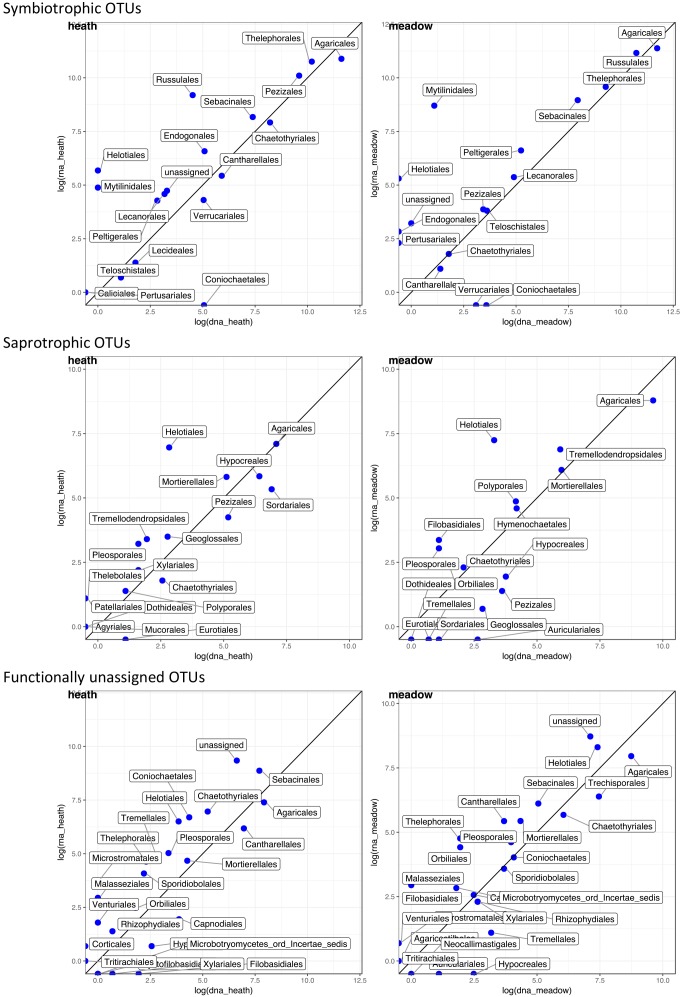
Correlation of rDNA- and rRNA-derived abundances of OTUs grouped in higher taxonomic rank (order) and divided into assigned trophic modes. Abundance data come from 9 pairs of coresponding rDNA and rRNA samples; data were log transformed. Data points above the line show orders which contributed more to rRNA than rDNA pool; and vice versa, data points beneath the line point out orders that contributed more to rDNA than rRNA pool.

Based on cumulative relative abundances of sequences, symbiotrophs were the dominant group in every combination of factors (metabarcoding template and vegetation type; Figure 3). The dominance in relative abundance of symbiotrophic reads was more pronounced in rDNA than rRNA, regardless of vegetation type (by 6.6% of the reads in the meadow and by 15.4% in the heath). Saprotrophs were more abundant in rRNA (by 6% of the reads in heath and 3.3% in meadow). rRNA harbored significantly more functionally unassigned sequences than rDNA, especially in heath where the difference was the highest (10.6% of reads). Similarly to saprotrophs, reads not assigned to any trophic mode, were twice as abundant in rRNA than rDNA-based results. We observed that an increase in relative number of reads from saprotrophic and unassigned trophic modes originated from overall higher richness, as well as highly expressed rRNA in a particular OTU (Figure 4).

### Taxonomic Groups

Although fungi in each trophic mode are functionally similar in the ecosystem, species can belong to distantly related fungal orders. For combination of trophic modes and vegetation types we detected taxonomic groups that might contribute in varying degree to a bias between rDNA and rRNA-derived results. Within each taxonomic group OTUs responded in different ways: some showed overexpressed rRNA, some were more abundant in rDNA and other OTUs did not change their abundance when rDNA and rRNA-derived results were compared. The most consistent overrepresentation of any taxonomic order in rDNA-results was observed in Agaricales in every combination of trophic mode and vegetation type, except saprotrophs in heath (Figure 4). There the numbers of Agaricales reads did not differ between rDNA and rRNA-derived results. Symbiotrophic reads overrepresented in rRNA belonged to Russulales and Thelephorales regardless of vegetation type, whereas overrepresentation of rRNA-derived sequences from Pezizales occurred in the heath. Saprotrophic reads that appeared more often in rRNA in both vegetation types were Helotiales, and additionally – only in the heath: Mortierellales and only in meadow: Tremellodendropsidales, whereas Sordariales and Hypocreales reads were more numerous in rDNA in the heath than in the meadow. Functionally unassigned reads in rRNA pool were predominantly unassigned taxonomically to order or higher rank in both vegetation types, whereas Sebacinales were found overexpressed only in the heath and Helotiales only in the meadow.

## Discussion

Similarities between rDNA and rRNA metabarcoding of microbial or cryptic species has received little attention in cold terrestrial environments. Low temperatures, often below 0°C, can slow microbial processes, including the decomposition of dead biomass. These cells remain in the soil and contribute to a pool of relic rDNA. Our case study contributes to understanding which types of analyses of sequences parsed in ecologically meaningful units may result in most discrepancy between the two metabarcoding templates. Moreover, we made an attempt to link both fungal functions and diversity, to pinpoint possible sources of differences in rDNA and rRNA-derived results from cold environments.

Our comparison between rDNA and rRNA metabarcoding templates unveiled no or little divergence in community composition, also when the sequences were divided into fungal trophic modes. The clustering according to vegetation type agrees with former studies, supporting the importance of a long-lasting interaction between fungal community structure and vegetation type (Chu et al., 2011; Shi et al., 2015). This general trend was also consistent for community composition of symbiotrophs and saprotrophs, demonstrating the fine-tuning of these functional groups with the vegetation type. Ordinations based on pathotrophs, the least represented group, both in number of OTUs and number of reads, did not show clear differences between vegetation types (as other trophic modes); this pattern may be due to their stochastic distribution in the soil (Bahram et al., 2016). We speculate that the strong impact of vegetation type can partly mask effects of other factors, such as metabarcoding template and snow fence treatment (Supplements 4, 5). Our findings suggest that a possible bias introduced by rDNA-based metabarcoding does not influence the main conclusions regarding community composition.

Symbiotrophs are usually the dominating fungal functional group in soils, also in the Arctic (Gardes and Dahlberg, 1996; Clemmensen et al., 2006; Mundra et al., 2016b), a trend supported by our study. Both the highest number of OTUs and the largest proportion of sequences belonged to symbiotrophs, especially Agaricales. Although dominating in both templates, a higher proportion of symbiotrophic reads that belong to Agaricales were detected in rDNA than in rRNA regardless of vegetation type. This may suggest that relatively more symbiotrophic rDNA originate from dead cells or extracellular rDNA (Carini et al., 2016). It is plausible that more symbiotrophic rDNA is retained in soil because Agaricales are simply more abundant than other fungi. On the other hand, symbiotrophic Thelephorales, Pezizales or Russulales might be overestimated when rRNA is used as an estimator for abundance. The observed higher proportions of saprotrophic reads in rRNA samples than in rDNA, suggest that saprotrophic OTUs produce relatively more rRNA, especially in Helotiales, hence are more active than the rDNA data would suggest. As we sampled only on one date it is not possible to tell whether data would be similar throughout the year or if there would be taxonomically specific responses to temporal dynamics within the tundra soil.

Fungi with functionally unassigned sequences comprised a substantial proportion of all heath sequences, based on rRNA. Unassigned sequences in this study originated mainly from novel organisms without any database matches but also from unresolved/ambiguous functions that change throughout fungal life cycle or due to changes in the environmental conditions (Figure 4). We argue that the taxonomically unresolved component of the fungal community contributes substantially to active fungal community and recommend looking into these unknown OTUs with unknown functions.

Differences in the explanatory power of environmental variables between rDNA and rRNA-based community compositions have been reported only in a few studies comparing outcomes from both metabarcoding templates (i.e., Barnard et al., 2013; Zhang et al., 2014; Lüneberg et al., 2018). Yet it seems important to understand which parameters are crucial for shaping the community composition. Our study confirmed that pH is an important edaphic variable (Bååth and Anderson, 2003; Rousk et al., 2010; Mundra et al., 2016a), which explained both rDNA and rRNA-derived community composition. However, the similarities between explanatory power of the most important edaphic variables between the two templates end here. Langenheder and Prosser (2008) found that resource availability (such as organic matter, nitrogen and carbon concentration) explained most variability within rRNA-based results from heterotrophic soil bacteria. Fungi are also heterotrophic organisms that rely on resource availability. Both bacteria and fungi enhance their growth rate and cellular capacity for protein synthesis when metabolically available nitrogen and carbon levels increase (for more on regulation: Broach, 2012; You et al., 2013). Effectively, this means that cells transcribe more rRNA in order to produce more ribosomes for protein synthesis, to use available resources more efficiently.

The level of expressed rRNA is not always equivalent to the level of growing and dividing cells. Instead, the increased number of rRNA may rather be a stress response for handling multiple stressors and in order to do so, cells transcribe more ribosomes than they would for growth without these stressors (Blazewicz et al., 2013). Contrary to some microbial dormant stages, such as bacterial spores, basidiospores of five species of fungi were shown not to contain rRNA (Van der Linde and Haller, 2013), implying that by using rRNA in our study we eliminate the contribution of not only dead cells, but also dormant stages of fungi. However, just before germination when the spores swell, the amount of rRNA increases rapidly and not proportionally to normal cellular growth (Moore et al., 2011), possibly influencing our results to some extent.

The relationship between the number of sequences originating from rDNA and rRNA is complex. The number of rDNA copies in a genome differs between organisms, also between fungal species (Torres-Machorro et al., 2010; Black et al., 2013; Das and Deb, 2015; Johnson et al., 2015). Copy numbers of rRNA (ribosomes) can differ depending on conditions and is a result of the synthesis and degradation rates (Blazewicz et al., 2013). However, by targeting the ITS fragments in this study, we eliminated the influence of ribosome degradation rates, since ITS is removed from the rRNA precursor prior to ribosome formation (Schoch et al., 2012). While relationships between cellular growth and rRNA can be measured for cultured organisms in carefully controlled laboratory conditions, it is not known how this ratio is maintained in a complex environment full of interactions and stressors. It is, however, clear that rDNA copy numbers vary less over time or in different conditions than the number of rRNA per cell, making rDNA a rather poor predictor of growth or approximation of biomass content (Blazewicz et al., 2013).

Changes of environmental and edaphic parameters can cause shifts in fungal community compositions or in fungal richness. Strong seasonality in environments, such as in the Arctic tundra, lead to temporal dynamics within fungal communities (Mundra et al., 2015a), which can respond differently to changing conditions depending on their function in the ecosystem (Mundra et al., 2016b). At the same time, cold conditions may delay decomposition or favor preservation of dead biomass (Conant et al., 2011; Ejarque and Abakumov, 2016). In these circumstances, microbial communities monitored only with rDNA-based marker genes reflect not only currently thriving microbes, but also these active in the past, even in a multidecade time frame (Yoccoz et al., 2012). Our study explored differences of tundra soil between total and active fungal communities at only one time point. A study of the temporal dynamics of rDNA and rRNA across all seasons would profoundly enhance our understanding of the possible seasonal differences in microbial community composition, especially after major changes in environmental conditions.

## Data Availability Statement

Sequencing data generated for this study is available online: 10.5281/zenodo.1462886. Detailed description of bioinformatics pipeline, mapping file for demultiplexing, environmental dataset, OTU table, taxonomic and functional assignments and R code generated for this study is available at https://github.com/magdawutkowska/Dead_or_alive.

## Author Contributions

MW analyzed the data and wrote the manuscript. AV planned, collected samples, processed samples, analyzed the data, discussed the results, and the manuscript. SM planned, collected samples, discussed the results, and the manuscript. EC designed the sampling site, discussed the results, and the manuscript. PE planned, financed, discussed the results, and wrote the manuscript.

## Conflict of Interest Statement

The authors declare that the research was conducted in the absence of any commercial or financial relationships that could be construed as a potential conflict of interest.

## Footnotes

1 https://github.com/indraniel/fqgrep

2 http://hannonlab.cshl.edu/fastx_toolkit/

## References

[B1] Anderson I. C. Parkin P. I. (2007). Detection of active soil fungi by RT-PCR amplification of precursor rRNA molecules. *J. Microbiol. Methods* 68 248–253. 10.1016/j.mimet.2006.08.005 17045683

[B2] Bååth E. Anderson T. H. (2003). Comparison of soil fungal/bacterial ratios in a pH gradient using physiological and PLFA-based techniques. *Soil Biol. Biochem.* 35 955–963. 10.1016/S0038-0717(03)00154-8

[B3] Bahram M. Kohout P. Anslan S. Harend H. Abarenkov K. Tedersoo L. (2016). Stochastic distribution of small soil eukaryotes resulting from high dispersal and drift in a local environment. *ISME J.* 10 885–896. 10.1038/ismej.2015.164 26394006 PMC4796928

[B4] Bálint M. Schmidt P. A. Sharma R. Thines M. Schmitt I. (2014). An Illumina metabarcoding pipeline for fungi. *Ecol. Evol.* 4 2642–2653. 10.1002/ece3.1107 25077016 PMC4113289

[B5] Barnard R. L. Osborne C. A. Firestone M. K. (2013). Responses of soil bacterial and fungal communities to extreme desiccation and rewetting. *ISME J.* 7 2229–2241. 10.1038/ismej.2013.104 23823489 PMC3806258

[B6] Barnes M. A. Turner C. R. (2016). The ecology of environmental DNA and implications for conservation genetics. *Conserv. Genet.* 17 1–17. 10.1007/s10592-015-0775-4

[B7] Bass D. Stentiford G. D. Littlewood D. T. J. Hartikainen H. (2015). Diverse applications of environmental DNA methods in parasitology. *Trends Parasitol.* 31 499–513. 10.1016/j.pt.2015.06.013 26433253

[B8] Bates D. Maechler M. Bolker B. Walker S. (2015). Fitting linear mixed-effects models using lme4. *J. Stat. Soft.* 67 1–48. 10.18637/jss.v067.i01

[B9] Bengtsson-Palme J. Ryberg M. Hartmann M. Branco S. Wang Z. Godhe A. (2013). Improved software detection and extraction of ITS1 and ITS2 from ribosomal ITS sequences of fungi and other eukaryotes for analysis of environmental sequencing data. *Methods Ecol. Evol.* 4 914–919. 10.1111/2041-210X.12073

[B10] Blaalid R. Kumar S. Nilsson R. H. Abarenkov K. Kirk P. M. Kauserud H. (2013). ITS1 versus ITS2 as DNA metabarcodes for fungi. *Mol. Ecol. Resour.* 13 218–224. 10.1111/1755-0998.12065 23350562

[B11] Black J. Dean T. Byfield G. Foarde K. Menetrez M. (2013). Determining fungi rRNA copy number by PCR. *J. Biomol. Tech.* 24 32–38. 10.7171/jbt.13-2401-004 23543828 PMC3523570

[B12] Blazewicz S. J. Barnard R. L. Daly R. A. Firestone M. K. (2013). Evaluating rRNA as an indicator of microbial activity in environmental communities: limitations and uses. *ISME J.* 7 2061–2068. 10.1038/ismej.2013.102 23823491 PMC3806256

[B13] Bohmann K. Evans A. Gilbert M. T. P. Carvalho G. R. Creer S. Knapp M. (2014). Environmental DNA for wildlife biology and biodiversity monitoring. *Trends Ecol. Evol.* 29 358–367. 10.1016/j.tree.2014.04.003 24821515

[B14] Broach J. R. (2012). Nutritional control of growth and development in yeast. *Genetics* 192 73–105. 10.1534/genetics.111.135731 22964838 PMC3430547

[B15] Buckeridge K. M. Grogan P. (2008). Deepened snow alters soil microbial nutrient limitations in arctic birch hummock tundra. *Appl. Soil Ecol.* 39 210–222. 10.1016/j.apsoil.2007.12.010

[B16] Caporaso J. G. Kuczynski J. Stombaugh J. Bittinger K. Bushman F. D. Costello E. K. (2010). Correspondence QIIME allows analysis of high- throughput community sequencing data Intensity normalization improves color calling in SOLiD sequencing. *Nat. Publish. Group* 7 335–336. 10.1038/nmeth0510-335 PMC3156573 20383131

[B17] Cardinale B. J. Duffy J. E. Gonzalez A. Hooper D. U. Perrings C. Venail P. (2012). Biodiversity loss and its impact on humanity. *Nature* 486 59–67. 10.1038/nature11148 22678280

[B18] Carini P. Marsden P. J. Leff J. W. Morgan E. E. Strickland M. S. Fierer N. (2016). Relic DNA is abundant in soil and obscures estimates of soil microbial diversity. *Nat. Microbiol.* 2:16242. 10.1038/nmicrobiol.2016.242 27991881

[B19] Cernansky R. (2017). Biodiversity moves beyond counting species. *Nature* 546 22–24. 10.1038/546022a 28569825

[B20] Chessel D. Dufour A. B. Thioulouse J. (2004). The ade4 package – I?: one-table methods. *R News* 4 5–10.

[B21] Chu H. Neufeld J. D. Walker V. K. Grogan P. (2011). The influence of vegetation type on the dominant soil bacteria, archaea, and fungi in a low arctic tundra landscape. *Soil Sci. Soc. Am. J.* 75:1756 10.2136/sssaj2011.0057

[B22] Clemmensen K. E. Michelsen A. Jonasson S. Shaver G. R. (2006). Increased ectomycorrhizal fungal abundance after long-term fertilization and warming of two arctic tundra ecosystems. *New Phytol.* 171 391–404. 10.1111/j.1469-8137.2006.01778.x 16866945

[B23] Conant R. T. Ryan M. G. Ågren G. I. Birge H. E. Davidson E. A. Eliasson P. E. (2011). Temperature and soil organic matter decomposition rates – Synthesis of current knowledge and a way forward. *Glob. Change Biol.* 17 3392–3404. 10.1111/j.1365-2486.2011.02496.x

[B24] Cooper E. J. Dullinger S. Semenchuk P. (2011). Late snowmelt delays plant development and results in lower reproductive success in the High Arctic. *Plant Sci.* 180 157–167. 10.1016/j.plantsci.2010.09.005 21421357

[B25] Costello M. J. (2015). Biodiversity: the known, unknown, and rates of extinction. *Curr. Biol.* 25 R368–R371. 10.1016/j.cub.2015.03.051 25942550

[B26] Creer S. Deiner K. Frey S. Porazinska D. Taberlet P. Thomas W. K. (2016). The ecologist’s field guide to sequence-based identification of biodiversity. *Methods Ecol. Evol.* 7 1008–1018. 10.1111/2041-210X.12574

[B27] Das S. Deb B. (2015). DNA barcoding of fungi using ribosomal ITS marker for genetic diversity analysis: a review. *Int. J. Pure Appl. Biosci.* 3 160–167. 22891635

[B28] Edgar R. C. (2010). Search and clustering orders of magnitude faster than BLAST. *Bioinformatics* 26 2460–2461. 10.1093/bioinformatics/btq461 20709691

[B29] Edgar R. C. (2016). UCHIME2: improved chimera prediction for amplicon sequencing. *BioRxiv* [preprint]. 10.1101/074252

[B30] Ejarque E. Abakumov E. (2016). Stability and biodegradability of organic matter from Arctic soils of Western Siberia: insights from13C-NMR spectroscopy and elemental analysis. *Solid Earth* 7 153–165. 10.5194/se-7-153-2016

[B31] Fodor E. (2011). Ecological niche of plant pathogens. *Ann. For. Res.* 54 3–21.

[B32] Fox J. Weisberg S. (2011). *An R Companion to Applied Regression*, 2nd Edn. Thousand Oaks, CA: Sage.

[B33] Gardes M. Dahlberg A. (1996). Mycorrhizal diversity in arctic and alpine tundra: an open question. *New Phytol.* 133 147–157. 10.1111/j.1469-8137.1996.tb04350.x

[B34] Gilichinsky D. A. Wagener S. Vishnevetskaya T. A. (1995). Permafrost microbiology. *Perm. Perigl. Process.* 6 281–291. 10.1002/ppp.3430060402

[B35] Goldberg C. S. Turner C. R. Deiner K. Klymus K. E. Thomsen P. F. Murphy M. A. (2016). Critical considerations for the application of environmental DNA methods to detect aquatic species. *Methods Ecol. Evol.* 7 1299–1307. 10.1111/2041-210X.12595

[B36] Hawksworth D. L. Lücking R. (2017). Fungal diversity revisited?: 2. 2 to 3. 8 million species. *Microbiol. Spect.* 5 1–17. 10.1128/microbiolspec.FUNK-0052-2016.Correspondence 28752818 PMC11687528

[B37] Huson D. H. Beier S. Flade I. Górska A. El-Hadidi M. Mitra S. (2016). MEGAN community edition – Interactive exploration and analysis of large-scale microbiome sequencing data. *PLoS Comput. Biol.* 12:e04957. 10.1371/journal.pcbi.1004957 27327495 PMC4915700

[B38] Ihrmark K. Bödeker I. T. M. Cruz-Martinez K. Friberg H. Kubartova A. Schenck J. (2012). New primers to amplify the fungal ITS2 region – Evaluation by 454-sequencing of artificial and natural communities. *FEMS Microbiol. Ecol.* 82 666–677. 10.1111/j.1574-6941.2012.01437.x 22738186

[B39] Johnson S. M. Carlson E. L. Pappagianis D. (2015). Determination of ribosomal DNA copy number and comparison among strains of coccidioides. *Mycopathologia* 179 45–51. 10.1007/s11046-014-9820-y 25322704

[B40] Kaiser C. Meyer H. Biasi C. Rusalimova O. Barsukov P. Richter A. (2007). Conservation of soil organic matter through cryoturbation in arctic soils in Siberia. *J. Geophys. Res. Biogeosci.* 112 1–8. 10.1029/2006JG000258

[B41] Kohler A. Kuo A. Nagy L. G. Morin E. Barry K. W. Buscot F. (2015). Convergent losses of decay mechanisms and rapid turnover of symbiosis genes in mycorrhizal mutualists. *Nat. Genet.* 47 410–415. 10.1038/ng.3223 25706625

[B42] Kõljalg U. Nilsson R. H. Abarenkov K. Tedersoo L. Taylor A. F. S. Bahram M. (2013). Towards a unified paradigm for sequence-based identification of fungi. *Mol. Ecol.* 22 5271–5277. 10.1111/mec.12481 24112409

[B43] Konopka A. (2009). What is microbial community ecology. *ISME J.* 3 1223–1230. 10.1038/ismej.2009.88 19657372

[B44] Kruskal J. B. (1964). Multidimensional scaling by optimizing goodness of fit to a nonmetric hypothesis. *Psychometrika* 29 1–27. 10.1007/BF02289565

[B45] Langenheder S. Prosser J. I. (2008). Resource availability influences the diversity of a functional group of heterotrophic soil bacteria. *Environ. Microbiol.* 10 2245–2256. 10.1111/j.1462-2920.2008.01647.x 18479445

[B46] Li R. Tun H. M. Jahan M. Zhang Z. Kumar A. Fernando D. (2017). Comparison of DNA-, PMA-, and RNA-based 16S rRNA Illumina sequencing for detection of live bacteria in water. *Sci. Rep.* 7:5752. 10.1038/s41598-017-02516-3 28720878 PMC5515937

[B47] Liu H. Økland T. Halvorsen R. Gao J. Liu Q. Eilertsen O. (2008). Gradients analyses of forests ground vegetation and its relationships to environmental variables in five subtropical forest areas, S and SW China. *Sommerfeltia* 32 1–196. 10.2478/v10208-011-0012-6

[B48] Louca S. Jacques S. M. S. Pires A. P. F. Leal J. S. Srivastava D. S. Parfrey L. W. (2016). High taxonomic variability despite stable functional structure across microbial communities. *Nat. Ecol. Evol.* 1:0015. 10.1038/s41559-016-0015 28812567

[B49] Lüneberg K. Schneider D. Siebe C. Daniel R. (2018). Drylands soil bacterial community is affected by land use change and different irrigation practices in the Mezquital Valley, Mexico. *Sci. Rep.* 8 1–15. 10.1038/s41598-018-19743-x 29362388 PMC5780513

[B50] Masella A. P. Bartram A. K. Truszkowski J. M. Brown D. G. Neufeld J. D. (2012). PANDAseq: paired-end assembler for illumina sequences. *BMC Bioinformatics* 13:31. 10.1186/1471-2105-13-31 22333067 PMC3471323

[B51] Moore D. Robson G. D. Trinci A. P. (2011). *21st Century Guidebook to Fungi.* Cambridge: Cambridge University Press 10.1017/CBO9780511977022

[B52] Morgado L. N. Semenova T. A. Welker J. M. Walker M. D. Smets E. Geml J. (2016). Long-term increase in snow depth leads to compositional changes in arctic ectomycorrhizal fungal communities. *Glob. Change Biol.* 22 3080–3096. 10.1111/gcb.13294 27004610

[B53] Morgner E. Elberling B. Strebel D. Cooper E. J. (2010). The importance of winter in annual ecosystem respiration in the High Arctic: effects of snow depth in two vegetation types. *Polar Res.* 29 58–74. 10.1111/j.1751-8369.2010.00151.x

[B54] Mundra S. Bahram M. Eidesen P. B. (2016a). Alpine bistort (*Bistorta vivipara*) in edge habitat associates with fewer but distinct ectomycorrhizal fungal species: a comparative study of three contrasting soil environments in Svalbard. *Mycorrhiza* 26 809–818. 10.1007/s00572-016-0716-1 27325524

[B55] Mundra S. Halvorsen R. Kauserud H. Bahram M. Tedersoo L. Elberling B. (2016b). Ectomycorrhizal and saprotrophic fungi respond differently to long-term experimentally increased snow depth in the High Arctic. *Microbiol. Open* 5 856–869. 10.1002/mbo3.375 27255701 PMC5061721

[B56] Mundra S. Bahram M. Tedersoo L. Kauserud H. Halvorsen R. Eidesen P. B. (2015a). Temporal variation of *Bistorta vivipara* -associated ectomycorrhizal fungal communities in the High Arctic. *Mol. Ecol.* 24 6289–6302. 10.1111/mec.13458 26547806

[B57] Mundra S. Halvorsen R. Kauserud H. H. Müller E. Vik U. Eidesen P. B. (2015b). Arctic fungal communities associated with roots of *Bistorta vivipara* do not respond to the same fine-scale edaphic gradients as the aboveground vegetation. *New Phytol.* 205 1587–1597. 10.1111/nph.13216 25483568

[B58] Nguyen N. H. Smith D. Peay K. Kennedy P. (2015). Parsing ecological signal from noise in next generation amplicon sequencing. *New Phytol.* 205 1389–1393. 10.1111/nph.12923 24985885

[B59] Nguyen N. H. Song Z. Bates S. T. Branco S. Tedersoo L. Menke J. (2016). FUNGuild: an open annotation tool for parsing fungal community datasets by ecological guild. *Fungal Ecol.* 20 241–248. 10.1016/j.funeco.2015.06.006

[B60] Ogram A. Sayler G. S. Gustln D. Lewis R. J. (1988). DNA adsorption to soils and sediments. *Environ. Sci. Technol.* 22 982–984. 10.1021/es00173a020 22195724

[B61] Oksanen J. Blanchet F. G. Friendly M. Kindt R. Lagendre P. McGlinn D. (2018). *Vegan: Community Ecology Package. R package version 2.5-1.* Available at: https://cran.r-project.org/package=vegan

[B62] Olofsson J. Ericson L. Torp M. Stark S. Baxter R. (2011). Carbon balance of Arctic tundra under increased snow cover mediated by a plant pathogen. *Nat. Clim. Change* 1 220–223. 10.1038/nclimate1142

[B63] Pedersen M. W. Overballe-Petersen S. Ermini L. Sarkissian C. Haile J. Hellstrom M. (2015). Ancient and modern environmental DNA. *Philos. Trans. R. Soc. B Biol. Sci.* 370:20130383. 10.1098/rstb.2013.0383 25487334 PMC4275890

[B64] R Core Team (2018). *R: A Language and Environment for Statistical Computing.* Vienna: Foundation for Statistical Computing.

[B65] Rognes T. Flouri T. Nichols B. Quince C. Mahé F. (2016). VSEARCH: a versatile open source tool for metagenomics. *PeerJ* 4:e2584. 10.7717/peerj.2584 27781170 PMC5075697

[B66] Rousk J. Bååth E. Brookes P. C. Lauber C. L. Lozupone C. Caporaso J. G. (2010). Soil bacterial and fungal communities across a pH gradient in an arable soil. *ISME J.* 4 1340–1351. 10.1038/ismej.2010.58 20445636

[B67] Saeki K. Kunito T. (2010). Adsorptions of DNA molecules by soils and variable-charged soil constituents GMOs. *Appl. Microbiol.* 1 188–195.

[B68] Saeki K. Kunito T. Sakai M. (2011). Effect of Tris-HCl buffer on DNA adsorption by a variety of soil constituents. *Microbes Environ.* 26 88–91. 10.1264/jsme2.ME10172 21487209

[B69] Schoch C. L. Seifert K. A. Huhndorf S. Robert V. Spouge J. L. Levesque C. A. (2012). Nuclear ribosomal internal transcribed spacer (ITS) region as a universal DNA barcode marker for Fungi. *Proc. Natl. Acad. Sci. U.S.A.* 109 1–6. 10.1073/pnas.1117018109 22454494 PMC3341068

[B70] Semenchuk P. R. Elberling B. Amtorp C. Winkler J. Rumpf S. Michelsen A. (2015). Deeper snow alters soil nutrient availability and leaf nutrient status in high Arctic tundra. *Biogeochemistry* 124 81–94. 10.1007/s10533-015-0082-7

[B71] Semenova T. A. Morgado L. N. Welker J. M. Walker M. D. Smets E. Geml J. J. (2016). Compositional and functional shifts in arctic fungal communities in response to experimentally increased snow depth. *Soil Biol. Biochem.* 100 201–209. 10.1016/j.soilbio.2016.06.001

[B72] Shelton A. O. O’Donnell J. L. Samhouri J. F. Lowell N. Williams G. D. Kelly R. P. (2016). A framework for inferring biological communities from environmental DNA. *Ecol. Appl.* 26 1645–1659. 10.1890/15-1733.1 27755698

[B73] Shi Y. Xiang X. Shen C. Chu H. Neufeld J. D. Walker V. K. (2015). Vegetation-associated impacts on Arctic tundra bacterial and microeukaryotic communities. *Appl. Environ. Microbiol.* 81 492–501. 10.1128/AEM.03229-14 25362064 PMC4277566

[B74] Soina V. S. Vorobiova E. A. Zvyagintsev D. G. Gilichinsky D. A. (1995). Preservation of cell structures in permafrost: a model for exobiology. *Adv. Space Res.* 15 237–242. 10.1016/S0273-1177(99)80090-8 11539231

[B75] Thomsen P. F. Willerslev E. (2015). Environmental DNA – An emerging tool in conservation for monitoring past and present biodiversity. *Biol. Conserv.* 183 4–18. 10.1016/j.biocon.2014.11.019

[B76] Timling I. Taylor D. L. (2012). Peeking through a frosty window: molecular insights into the ecology of Arctic soil fungi. *Fungal Ecol.* 5 419–429. 10.1016/j.funeco.2012.01.009

[B77] Torres-Machorro A. L. Hernández R. Cevallos A. M. López-Villaseñor I. (2010). Ribosomal RNA genes in eukaryotic microorganisms: witnesses of phylogeny? *FEMS Microbiol. Rev.* 34 59–86. 10.1111/j.1574-6976.2009.00196.x 19930463

[B78] Van der Linde S. Haller S. (2013). Obtaining a spore free fungal community composition. *Fungal Ecol.* 6 522–526. 10.1016/j.funeco.2013.10.001

[B79] White T. J. Bruns S. Lee S. Taylor J. (1990). “Amplification and direct sequencing of fungal ribosomal RNA genes for phyologenetics,” in *PCR Protocols: A Guide to Methods and Applications*, eds Innis M. A. Gelfand D. H. Sninsky J. J. (New York, NY: Academic Press Inc.).

[B80] Yoccoz N. G. Bråthen K. A. Gielly L. Haile J. Edwards M. E. Goslar T. (2012). DNA from soil mirrors plant taxonomic and growth form diversity. *Mol. Ecol.* 21 3647–3655. 10.1111/j.1365-294X.2012.05545.x 22507540

[B81] You C. Okano H. Hui S. Zhang Z. Kim M. Gunderson C. W. (2013). Coordination of bacterial proteome with metabolism by cyclic AMP signalling. *Nature* 500 301–306. 10.1038/nature12446 23925119 PMC4038431

[B82] Zhang Y. Zhao Z. Dai M. Jiao N. Herndl G. J. (2014). Drivers shaping the diversity and biogeography of total and active bacterial communities in the South China Sea. *Mol. Ecol.* 23 2260–2274. 10.1111/mec.12739 24684298 PMC4230472

