# Supplementary material for: Dead or Alive; or Does It Really Matter? Level of Congruency Between Trophic Modes in Total and Active Fungal Communities in High Arctic Soil
Source: Front Microbiol. 2019 Jan 8;9:3243. doi: 10.3389/fmicb.2018.03243 (PMC6333106; doi:10.3389/fmicb.2018.03243)
Supplement: Supplementary file 1 [file Data_Sheet_1.docx]

Supplementary Material

**Dead or alive; or does it really matter? Level of congruency between trophic modes in total and active fungal communities in High Arctic soil.**

Magdalena Wutkowska* ^1, 2^, Anna Vader ^1^, Sunil Mundra ^3^, Elisabeth J. Cooper ^2^, Pernille B. Eidesen ^1^*** Correspondence:** Corresponding Author: magdalena.wutkowska@unis.no; [magda.wutkowska@gmail.com](mailto:magda.wutkowska@gmail.com)

# Supplementary Figures and Tables

Supplement 1a | Experimental setup and design of the study. Twelve fences were erected, six on each of meadow and heath vegetation. The fences/ambient areas were arranged into blocks of three across the landscape, within an area of approx. 1.5-2.5 km. Heaths were dominated by *Cassiope tetragona* and had faster-draining stony soils and more undulating topography than the flatter meadows dominated by *Salix polaris* and *Luzula arcuata ssp. confusa* (Morgner et al., 2010). Snow depth and resulting date of snowmelt were manipulated using fences placed perpendicular to the winter wind direction. Beside each fence an unmanipulated area was designated with ambient snow conditions. Ambient regimes had 10-35cm snow in winter, whereas deep regimes had up to 150cm snow, and melted out ca. 17 days later (Semenchuk *et al*., 2013).

Supplement 1b | Satellite image of the snow fence experimental setup in Adventdalen, Svalbard. A1-B3 fences are located in two vegetation types: heath and in meadow.


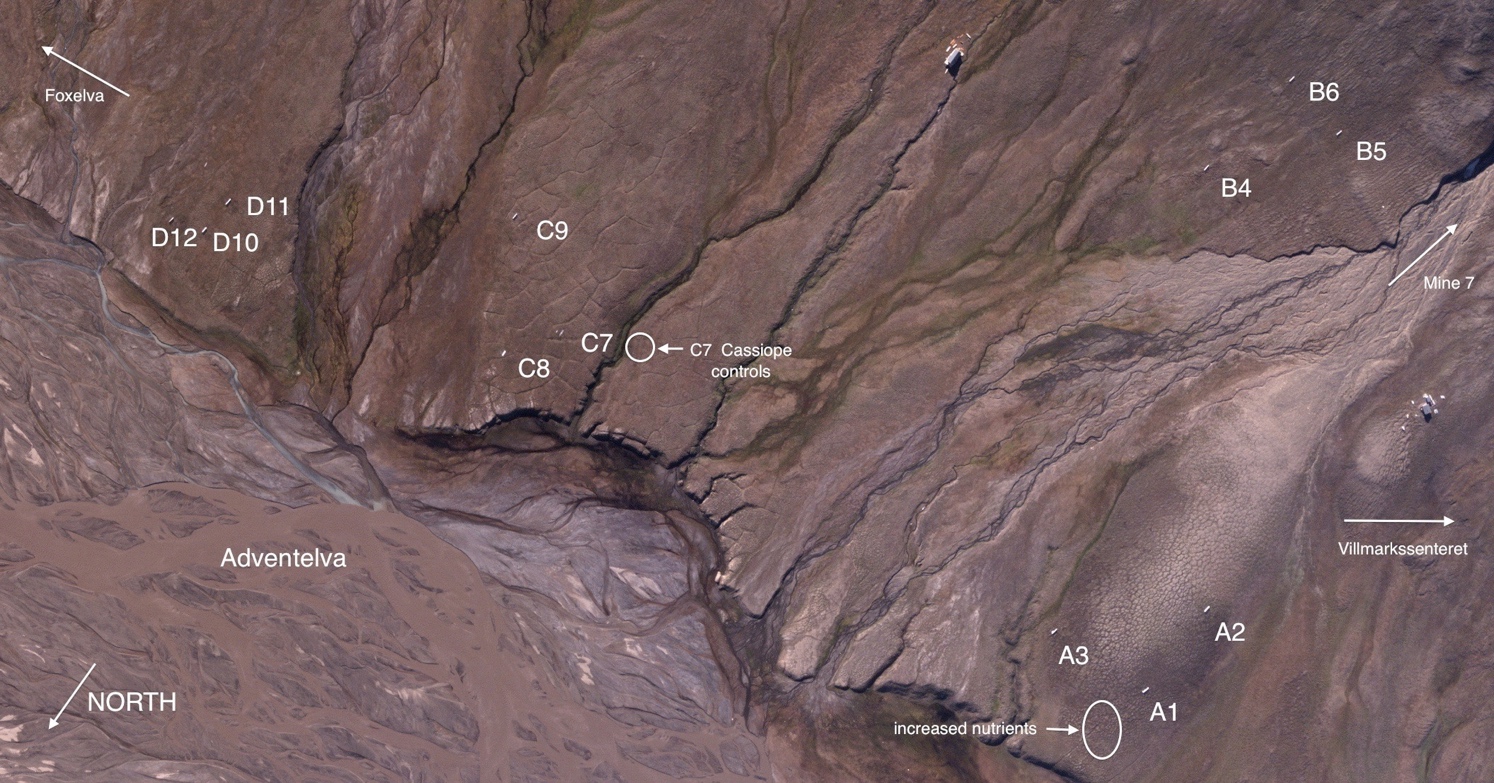


Supplement 2 | Boxplots representing differences in edaphic variables among ambient (A) and deep (D) snow regimes in two vegetation types heath (H) and meadow (M).


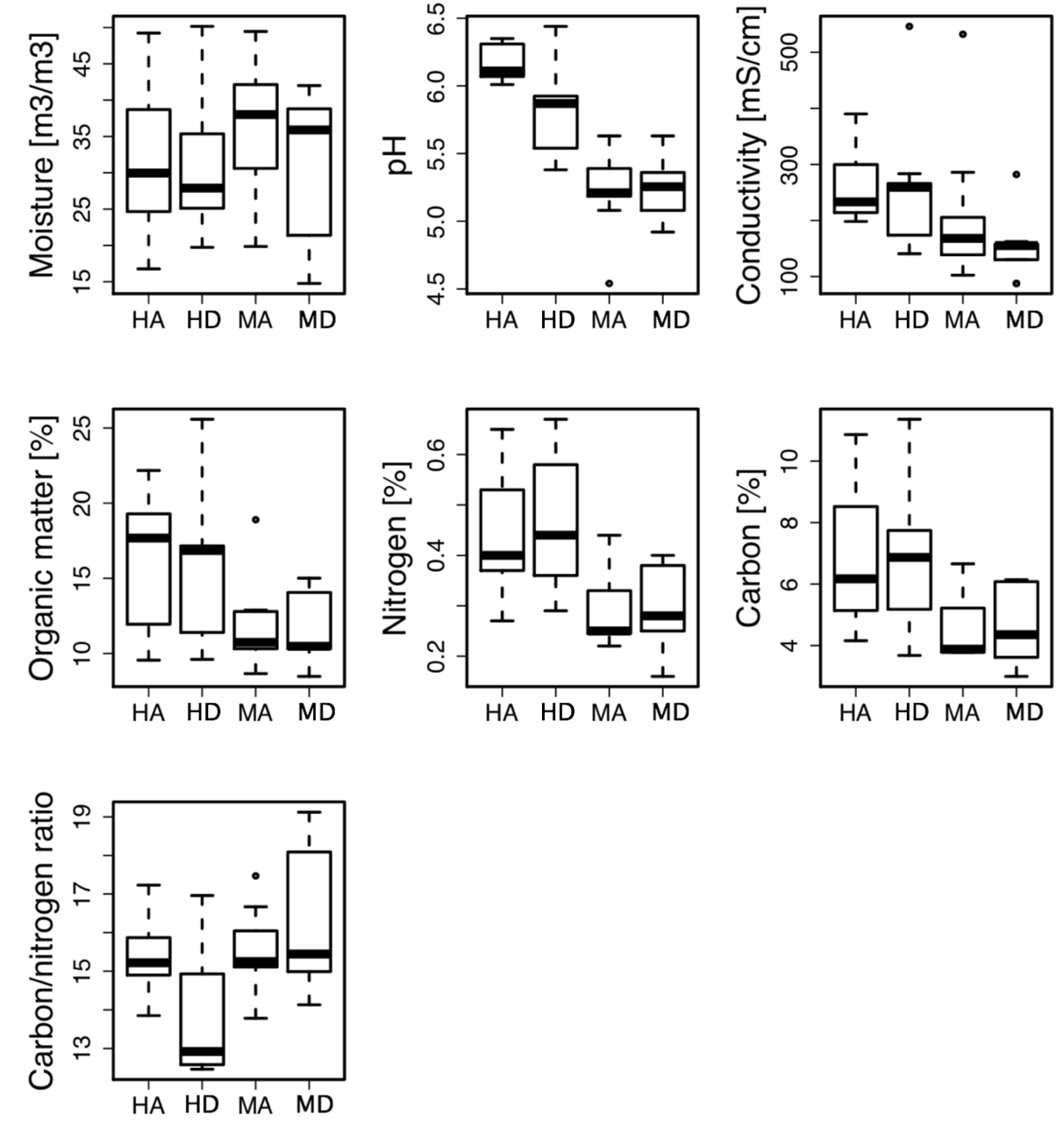


Supplement 3 | Summary information of the dataset.

|  | all OTUs (n=42) |  |  | rDNA_OTUs (n=23) |  |  | rRNA_OTUs (n=19) |  |  |
| --- | --- | --- | --- | --- | --- | --- | --- | --- | --- |
|  | total number of OTUs | mean no of reads per sample ± SD | percentage of total number of reads | total number of OTUs | mean no of reads per sample ± SD | percentage of total number of reads | total number of OTUs | mean number of reads per sample ± SD | percentage of total number of reads |
| pathotroph | 34 | 102±215 | 0.2% | 27 | 95±121 | 0.2% | 22 | 111±296 | 0.2% |
| saprotroph | 105 | 2460±4428 | 5.8% | 83 | 1528±3356 | 3.6% | 83 | 3588±5334 | 8.4% |
| symbiotroph | 288 | 35820±7787 | 84.3% | 237 | 37981±5236 | 89.4% | 227 | 33203±9561 | 78.2% |
| unassigned | 410 | 4105±4909 | 9.7% | 309 | 2884±4302 | 6.8% | 306 | 5585±5298 | 13.1% |
| sum | 837 |  |  | 656 |  |  | 638 |  |  |

Supplement 4 | Global non-dimensional scaling of all 42 samples plotted according to template (rDNA/rRNA), vegetation type (H – heath, M - meadow) and snow regime (A – ambient snow regime, D – deep snow regime introduced by snow fences). Figure is based on presence-absence table.

Supplement 5 | OTU richness per experimental factor: template, vegetation type and snow regime. Red lines connects mean values between levels of each factor (with outliers).
